# Ecological security assessment of Yunnan Province, China in the context of Production–Living–Ecological space division

**DOI:** 10.1002/ece3.70131

**Published:** 2024-08-09

**Authors:** Fang Liu, Qian Zhang, Jinliang Wang, Yuexiong Liu, Wanbin Wang, Sen Li

**Affiliations:** ^1^ Faculty of Geography Yunnan Normal University Kunming China; ^2^ Yunnan Academy of Ecological and Environmental Sciences Kunming Yunnan China; ^3^ Key Laboratory of Resources and Environmental Remote Sensing for Universities in Yunnan Kunming China; ^4^ Center for Geospatial Information Engineering and Technology of Yunnan Province Kunming China

**Keywords:** ecological security, multiscale, PSR model, territorial space zoning

## Abstract

With the rapid development of population, society and economy, human activities have caused serious adverse impacts on the environment, ecosystems and landscape patterns over the long term. In order to address the series of impacts of human activities on the environment, territorial space and resource use, the study of Production–Living–Ecological Space (PLES) and ecological security have all become academic frontiers in the field of sustainable development. In this study, we applied multi‐source data and GIS technology to construct an ecological security evaluation model based on the results of PLES delineation and the Pressure‐State‐Response (PSR) framework, and carried out the three‐period PLES ecological security evaluation for 2000, 2010 and 2020 at the county and grid scales in Yunnan Province. The PLES pattern in Yunnan Province is dominated by ecological space, which accounts for 75%, followed by 23% of production space, with ecological space shrinking from 2000 to 2020. Ecological security in ecological space and living space shows an improving trend from 2000 to 2020. The ecological security of production space improved in 2010 compared to 2000 but then showed a decreasing trend in 2020. Ecological security in ecological space shows that north‐western and southern Yunnan is safer than central Yunnan, while ecological security in living space is safer in central Yunnan, and ecological security in production space is better in southern Yunnan than in northern Yunnan. Comparison with related research results shows that the ecological security evaluation results of PLES in Yunnan Province in this study are scientific and reasonable. The ecological security evaluation model of PLES constructed in this study solves the problem of complex and incomplete ecological security evaluation indexes in the past, and the results of the study are more refined and precise, which provides new ideas for the study of regional ecological security.

## Abstract 2

Based on the functional attributes of land, the evaluation model of ecological security of Production–Living–Ecological space was constructed. Yunnan Province has the largest proportion of ecological space at 75%, but it has been shrinking in the last 20 years. In the past 20 years, the ecological security of ecological space and living space in Yunnan Province has shown an improvement trend, while production space has risen and then declined. This study analyses ecological security at different scales so that it can better reveal its spatial heterogeneity.

## 1 INTRODUCTION

Land space is a complex geographical space, including different themes such as land resources, water resources, mineral resources, ecological environment, and socio‐economic (Jin et al., 2021). The concept of multifunctionality has been gradually introduced into land use research from geography, and the 2001 EU SENSOR project recognised that multifunctionality in land use includes social, economic and environmental functions. The process of economic and social development of countries in the land use planning zoning has a common law, are from the survival of the dominant agricultural space development gradually shifted to the life of the dominant industrial space, and finally is the use of oriented to quality‐oriented ecological space protection and development (Cai et al., 2018). This study considers the national territory space as a complex system with multiple functions, and PLES, which is short for production space, living space and ecological space, is a category division from the perspective of multifunctionality of the national territory space. The existing research‐related content of the PLES includes the concept, formation mechanism, multi‐functional calculation, identification method, spatiotemporal pattern evolution and characteristics, coupling coordination state evaluation and spatial pattern optimisation, etc. (Cui et al., 2022; Lin et al., 2022; Wang et al., 2022; Zhao et al., 2021). The content of research has changed from theoretical exploration to practical application and is becoming increasingly rich. The research cycle has evolved from a single period to different periods to a long time series, and the evolution of spatial and temporal patterns has been gradually deepened. Currently, PLES identification methods are divided into two categories, namely, the subsumption and classification method and the quantitative measurement method. The quantitative measurement method is to conduct a comprehensive evaluation by constructing a PLES evaluation model and indicator system, and to formulate spatial superposition rules to achieve the identification of PLES. The subsumption classification method is based on the current land use data and generates the correspondence between land use types and PLES based on the a priori knowledge of experts, and the land use types are directly subsumed to identify PLES. The existing research on PLES in the long time series and the medium and macro scope is relatively scarce, and there is little research on the combination of PLES and ecological security.

The irrational exploitation and utilisation of natural ecosystems by human beings is increasing, leading to the over‐consumption of resources and triggering a large number of ecological and environmental problems. The world is facing environmental problems such as the degradation of ecosystems, the loss of biodiversity, the intensification of land desertification and soil erosion, and the pollution of water, gas and soil. This is exerting enormous pressure on the Earth's limited resources, ecology, and environmental security, leading to serious ecological crises and disasters, and threatening the security of human beings themselves, as well as jeopardising regional, national, and even global ecological security and sustainable development (Fu et al., 2012). The United Nations launched the 2030 Agenda for Sustainable Development at the 2015 Sustainable Development Summit, which covers 17 goals, including clean water and energy, sustainable cities and communities, and climate action, and calls for global development to move towards the Sustainable Development Goals (SDGs) (Dong & Zhang, 2016). Therefore, ecological security has become an integral part of national security and an important element of regional and national sustainable development research (Liu & Chang, 2015). Ecological security evaluation research is proposed to cope with the above problems and is an important way to cope with global environmental problems and realise sustainable development of human beings. There have been more achievements in ecological security evaluation, but there still exists the problem that the evaluation index system is complicated, difficult to quantify, not comprehensive, and there is no unified standard, which also leads to the fact that different regions or different research institutes may use different evaluation methods and standards, and the results are less comparable (Du et al., 2013). Therefore, there is still a need for an in‐depth exploration of the evaluation model in ecological security evaluation research, which can be carried out in terms of choosing suitable evaluation methods according to different research areas and different research purposes, making the final evaluation results more accurate and avoiding mutual interference. The ecological security evaluation based on the production, living and ecological attributes, and characteristics of the land space has received little attention and research. Because of the different ecological and environmental problems in different land function areas, the ecological security evaluation of PLES is important for promoting sustainable development, protecting the ecological environment, improving the quality of human habitat, and realising the harmonious development of the economy and society. Accordingly, more attention should be paid to the ecological security evaluation of PLES.

Yunnan Province is an important ecological barrier in the southwest of China and is located in the Sichuan–Yunnan Ecological Barrier Zone in the National Main Functional Area Plan, as well as an important ecological security barrier in the upper reaches of the Yangtze River. As China's southwest ecological security barrier area, Yunnan bears the strategic task of maintaining regional, national and even international ecological security, and studies related to the evaluation of ecological security in Yunnan Province have consequently become the focus and hotspot of research (Mo & Xiao, 2016; Zhu et al., 2017). Yunnan is also the headwaters and source of four international rivers, namely the Mekong, Irrawaddy, Nu and Red Rivers, and the ecological changes and transboundary impacts of the international rivers, as well as geo‐cooperation and maintenance of security, have attracted wide international attention, making it a hotspot for cross‐cutting researches in the disciplines of geography, ecology and geopolitics (He et al., 2014). In order to implement the requirements of the national ecological civilisation construction and actively integrate into the development strategy of the Yangtze River Economic Belt, Yunnan Province has in recent years set the important strategic goal of “striving to be the leader of the national ecological civilisation construction”. The Yunnan Provincial Land Space Plan also proposes the construction of an ecological security pattern of “three screens and two belts” to ensure the maintenance of regional ecosystem stability and ecological security.

In this study, through multi‐source data and GIS technology, we constructed an ecological security evaluation index system of PLES based on the results of the division of PLES using the PSR framework and evaluated the ecological security of PLES at the county and grid scales in Yunnan Province in the three phases of 2000, 2010 and 2020. The study of ecological security evaluation in Yunnan Province based on the PLES division is conducive to the study of the maintenance of China's ecological security pattern, which is of great significance to the realisation of the adjustment and optimisation of the spatial pattern of the national territory and the management, and is the practical significance of Yunnan Province in guiding the construction of the Southwest China Ecological Security Barrier Zone. The study provides path support for regional territorial spatial control and the realisation of ecological security and provides decision‐making support for regional and global ecological security and sustainable development, which is of great international and domestic political, economic, social, and environmental significance.

## 2 RESEARCH AREA AND DATA SOURCE

### 2.1 Study area

**FIGURE 1:**
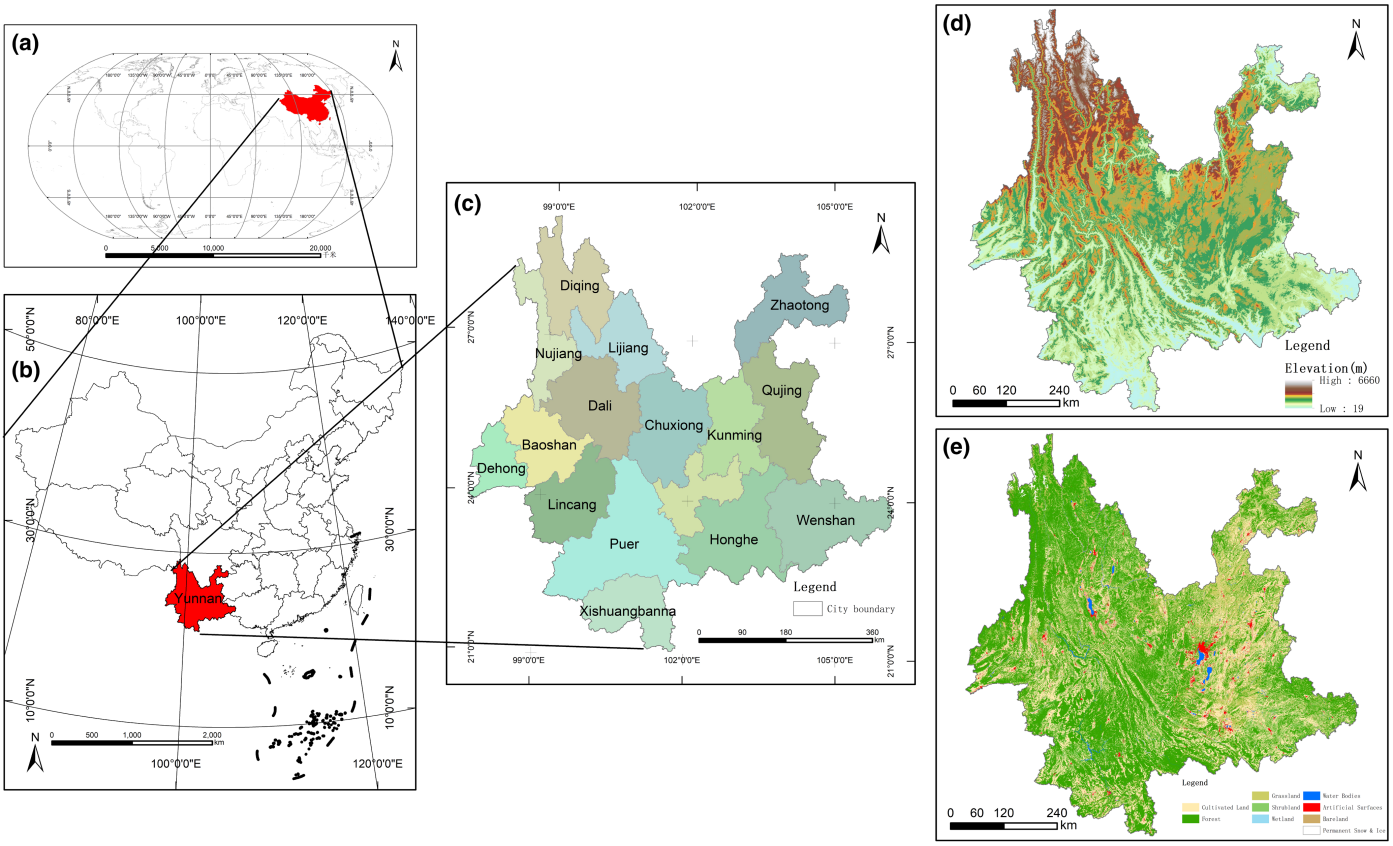
Map of Yunnan Province, China: Geographical location and basic information of Yunnan Province. (a) Global location, (b) national location, (c) administrative divisions of Yunnan Province, (d) topography of Yunnan Province and (e) land use in Yunnan Province in 2020.

Yunnan Province is located in the south‐western part of China, between longitude 97°31′–106°11′ East and latitude 21°8′–29°15′ North, with a total land area of 394,100 km^2^. It is bordered by Guangxi and Guizhou provinces in the east, Sichuan province in the north, Tibet in the northwest, Myanmar in the west, and Laos and Vietnam in the south and southeast, respectively, and is China's window and gateway to Southeast Asia and South Asia, and the location of the study area is schematically shown in Figure 1. The terrain is high in the northwest and low in the southeast, with an average elevation of about 2000 m, with mountains accounting for 94% of the total. The climate is dominated by the monsoon climate of the subtropical plateau, and due to the complex topography and large vertical height difference, the stereoscopic climate is characterised by remarkable features and diverse types. The unique geomorphology and rich climatic features have given rise to rich ecosystem types and a unique biodiversity system, with an extremely rich distribution of plants and animals, making it a key biodiversity area in the world. The total multi‐year average water resources is 221 billion cubic metres, which is the third largest in China. There are more than 40 plateau lakes, with a water area of about 1100 km^2^, and a total water storage capacity of 148.019 billion cubic metres. The province has 16 states and 129 counties, with a resident population of 46.93 million in 2022, GDP in 2022 was 2895.420 billion yuan, with a per capita disposable income of 26,937 yuan.

### 2.2 Data sources

The data used in the study mainly include MODIS data, DEM data, meteorological element data, land use data, ecological environment monitoring data, and socio‐economic statistics. Among them, the drought data and geohazard data come from the previous research results of the group (Nong et al., 2021; Yu et al., 2020). The specific sources of information are shown in Table 1.

**TABLE 1:** Data sources and introduction.

DATA	Type	Data content	Year	Resolution	Data sources
MODIS	Raster	Multi‐band remote sensing imagery	2000, 2010, 2020	500 m	http://www.gscloud.cn
DEM	Raster	Digital Elevation Model (DEM) ASTER GDEM V2	–	30 m	http://www.gscloud.cn
Temperatures	Raster	Monthly average near‐surface temperature	2000, 2010, 2020	1000 m	http://www.geodata.cn
Precipitation	Raster	Monthly precipitation	2000, 2010, 2020	1000 m	http://www.geodata.cn
River system	Vector	Waterline and water surface elements with lakes, reservoirs, and rivers	2020	1:100 million	http://mulu.tianditu.gov.cn
Land use/cover change data	Raster	30‐m global surface cover data with 10 primary land‐use types	2000, 2010, 2020	30 m	http://www.globallandcover.com
Environmental quality data	Documents	Including surface water environmental quality monitoring data	2000, 2010, 2020	–	Ecological Environment Quality Report
Administrative subdivision	Vector	County administrative boundaries across the province	2020	1:100 million	http://mulu.tianditu.gov.cn
GDP	Raster	Gross GDP within the kilometre grid in million yuan per square kilometre	2000, 2010, 2020	1000 m	https://www.resdc.cn
Population density	Raster	Population per square kilometre	2000, 2010, 2020	1000 m	https://www.worldpop.org/
Rode	Vector	Includes road, rail and transportation infrastructure elements	2000, 2010, 2020	1:100 million	http://www.geodata.cn
Soil organic	Raster	Content of organic matter in soil	2020	1000 m	http://data.tpdc.ac.cn
PM2.5	Raster	Annual ground‐level fine particulate matter (PM2.5) in μg/m^3^ (van Donkelaar et al., 2021)	2000, 2010, 2020	1000 m	https://sites.wustl.edu/acag/datasets/surface‐pm2‐5/

### 2.3 Data pre‐processing

Projection and resampling. All raster and vector data were uniformly projected to WGS84 coordinates, and all raster data were resampled to a 1 km × 1 km resolution grid according to the nearest neighbour method. Spatialisation of document data. For the statistical yearbook, water resources bulletin, environmental quality bulletin, and other document data, all kinds of document data are spatialised based on the administrative area vector data of Yunnan Province, and the water environmental quality will be analysed by combining the monitoring points and DEM for watershed analysis, to obtain the spatialised environmental quality data. The data spatialisation results are stored as raster data with uniform resolution and projection.

## 3 METHODS

### 3.1 Technical route

**FIGURE 2:**
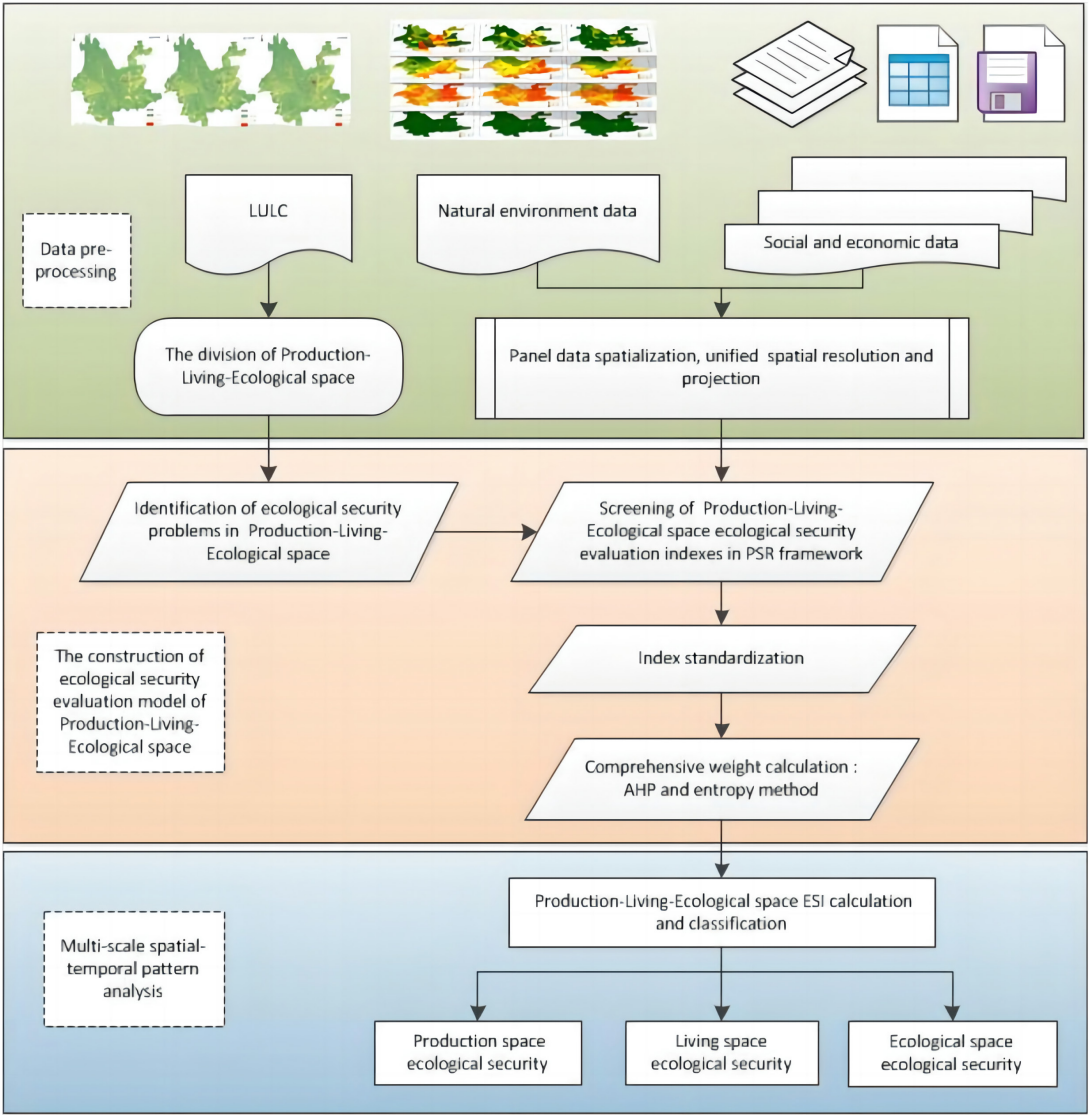
Technical route of the research.

In this study, based on multi‐source remote sensing data, land use, landscape ecology and PSR framework model, we explored the PLES spatial zoning in Yunnan Province, and based on the results of the zoning, we proceeded to establish a diagnostic model of ecological security in the zoning, and carried out the ecological security evaluation of the PLES respectively. Determine the ecological security objectives of PLES based on the analysis of the dominant and superior functions of each district. Analyse the main pressures, current status and response regulation measures facing the ecological security of each district under the PSR framework, and construct an evaluation model based on the ecological security objectives of PLES. The PSR model is widely used but its disadvantage is its strong dependent relationship, which can lead to a certain degree of overlap or redundancy in the selected indicators (Lai et al., 2022). According to the existing research results and indicator relevance and redundancy analysis, the screening and justification of indicators and the selection of specific indicators to complete the construction of the evaluation model. According to the existing research results and the analysis of indicator relevance and redundancy, the screening and demonstration of indicators are carried out. After determining the indicator system, the indicator standardisation method and weight calculation method were selected to complete the construction of the ecological security evaluation model of PLES, and finally the grading method of the ecological security evaluation results was selected to determine the ecological security level, and the technical route of the research is as follows (Figure 2).

### 3.2 Identification and classification of PLES

PLES spatial identification and classification is the basis for studying its spatiotemporal evolution characteristics, and the mainstream PLES identification methods can be categorised into the quantitative top‐down measurement method and the subsumed bottom‐up classification method (Liu et al., 2017). The quantitative measurement method is a comprehensive evaluation through a preset system of PLES spatial indicators to realise the quantitative identification of production, living and ecological spaces, but the amount of data required is large, and it is difficult to carry out multi‐subject integration and multi‐scale expression (Cui et al., 2022). The subsumption classification method is based on the correspondence between land use types and PLES, and the classification of PLES is carried out using this identified correspondence to realise the effective connection between PLES and land use classification. This method is concise, easy to operate and highly generalisable, and has the advantages of easy operation and generalisability, as it can quickly identify the distribution characteristics on the volume, and seamlessly connect with the land classification results for easy administrative management. The method is based on the current land use data, generating the correspondence between land classes and PLES based on the expert a priori knowledge, and identifying PLES by direct summarisation of land classes.

In this study, following the principle of from bottom to top and functional hierarchy, a classification system of PLES based on land use classification was established by Lin et al. (2021) (Table 2), which delineated the PLES of Yunnan Province in 2000, 2010 and 2020 at the scale of 1‐km grid (Lin et al., 2021).

**TABLE 2:** Land classification system and basis for PLES land use.

PLES	PLES categories	Corresponding land use type
Production space	Agricultural production space	Drylands, paddy fields
Living space	Urban living space and rural living space	Artificial surface
Ecological space	Forest ecological space, grassland ecological space, wetland ecological space and other ecological spaces	Woodlands, grasslands, shrublands, wetlands, water bodies, tundra and bare ground

### 3.3 Evaluation modelling of ecological security in PLES

#### 3.3.1 PLES ecological security evaluation index system

Production space is mainly an agricultural production area, its main function is agricultural production, industrial construction, tourism and finance, and other production activities, through the production activities for people's lives to provide a variety of agricultural products and services, is the process of human transformation of nature and the exploitation of nature (Xu, 2017). The Chinese Institute of Agricultural Resources and Zoning summarises the functions of agriculture into four major categories: supply of agricultural products, employment and security, ecological regulation, and cultural heritage and leisure (Tao & Luo, 2004). Specific functions are rural economic development and employment security, maintenance of cultural heritage and legacy, biodiversity, recreation and tourism, soil and water health, landscape, carbon sinks, food quality and security, and ecological and environmental protection (Zhu, 2011). Considering comprehensively the farming conditions, environmental pressure and resource inputs in Yunnan Province, we will construct an evaluation index system for the ecological security of production space.

Living space is a space to meet the needs of people's life and residence, and to provide carrying functions for human survival and development, with the dual safeguard function of material level and spiritual level, providing consumption, living, entertainment, and leisure environment for human daily life, which is the basis for people's survival and development (Fu, 2021; He, 2020). The evaluation index system of ecological security of living space is constructed considering land resources, environmental conditions and urban development factors in Yunnan Province.

Ecological space provides ecosystem services and ecological environment regulation as the dominant function but also has a certain cultural supply and support services, reflecting the natural background conditions of the region (Fu et al., 2012; Xie et al., 2015). Ecosystem service functions mainly include water conservation, soil and water conservation, biodiversity maintenance, wind and sand control, etc., ecosystem regulation functions mainly include landscape maintenance, ecological protection, environmental purification, climate regulation, carbon storage and sinks, and cultural provisioning and support services include the provision of ecological products, etc. (Li & Gao, 2019; Wang et al., 2012). The ecological space and ecological security evaluation index system considers factors such as ecosystem stability, ecosystem vulnerability, geologic risk and landscape pattern in Yunnan Province (Fang et al., 2021; Han et al., 2022; Liu et al., 2022).

The evaluation index system of ecological security in PLES constructed in this study is divided into three sub‐systems of PSR, with 13 indexes in the evaluation model of ecological security in living space, 10 indexes in the evaluation model of ecological security in production space and 11 indexes in the evaluation model of ecological security in ecological space, which is a total of 34 indexes (Table 3).

**TABLE 3:** Table of indicators for assessing the ecological security of PLES.

Standardised layer	Ecological security of living space			Ecological security of production space			Ecological security of ecological space		
	Indicator layer	Properties	Descriptions	Indicator layer	Properties	Descriptions	Indicator layer	Properties	Descriptions
Pressures (P)	Population density LSP1	−	Population per unit of land area, Unit square kilometres per person	Primary industry output per unit of land area PSP1	+	Gross value of agricultural, forestry, livestock, and fisheries production/area planted with crops	GDP per capitaESP1	−	Total output/total population
	Construction land area per capita LSP2	+	Total area of construction land/total population	Cultivated land area per capitaPSP2	−	Cultivated land area/population	Human Disturbance ESP2	−	Intensity of impact of human activities on ecosystems
	Emissions of pollutants LSP3	−	The volume of industrial wastewater discharged from enterprise outfalls	Intensity of fertiliser usePSP3	−	Fertiliser application/area planted with crops	Geologic hazard sensitivity ESP3	−	Density of geohazard sites
				Pesticide application intensityPSP4	−	Pesticide application/area planted with crops			
				Natural disaster ratePSP5	−	The probability of natural disasters such as drought			
State(S)	Air quality LSS1	−	Annual average PM2.5 concentration	Annual precipitation PSS1	+	Annual precipitation	Vegetation cover ESS1	+	Vegetation cover calculated based on remote sensing data
	Quality of the water environment LSS2	−	Annual water environment quality	Conditions of accumulated temperaturePSS2	+	The cumulative sum of daily average temperatures during the year	Biological abundance index ESS2	+	Differences in the number of living species in different ecosystem types
	Green space per capitaLSS3	+	The average area of parkland per person	Soil organic contentPSS3	+	Soil organic matter content	PD ESS3	−	Patch density
	Number of medical facility beds per million population LSS4	+	Beds in medical institutions per million inhabitants				SHEI ESS4	+	Shannon diversity Index
							CONTIG ESS5	−	Connectivity index
	Road densityLSS5	+	The ratio of the total length of roads to the area of the region				Ecosystem vulnerability index ESS6	−	Integrating indices of ecological sensitivity, ecological resilience, and ecological stress
Response(R)	Urban sewage treatment rate LSR1	+	The ratio of sewage volume treated to the total amount discharged	Effective irrigated area ratio PSR1	+	Effective irrigated area/cultivated area	Percentage of area under protectionESR1	+	Proportion of protected surface in the region to the total area of the region
	Industrial solid waste disposal rate LSR2	+	Comprehensive utilisation of industrial solid waste as a percentage of the amount generated	Per capita disposable income of farmersPSR2	+	Farmers' discretionary income	Local general public budget expenditure per capita ESR2	+	General budget expenditure/total population
	Number of students enrolled in middle school per million population LSR3	+	General middle school enrolment per million population						
	Public budget expenditure per capitaLSR4	+	Local budget expenditure/total population						
	GDP per capita LSR5	+	The ratio of GDP to population						

#### 3.3.2 Standardisation and weighting of indicators

Due to the different data types and nature of the evaluation indicators, including both quantitative and non‐quantitative indicators, there is a lack of comparability, which makes it impossible to directly carry out ecological security evaluation calculations. To eliminate the impact of these differences on the evaluation results, and to make the evaluation calculations more scientific and the results credible, it is necessary to carry out a non‐quantitative processing of all the indicators. Commonly used processing methods in existing studies include extreme difference standardisation, efficacy coefficient method, expert method, linear method, etc., and the extreme difference standardisation method has a better effect on the dimensionless processing of indicators, so the extreme difference standardisation method is selected in this study (Lu et al., 2018). The calculation method and process are as follows:

According to the role of the evaluation model indicators of ecological security can be divided into positive and negative indicators.

For positive indicators, which means that the larger the value of the indicator the better, the standardised formula for positive indicators is as follows:

(1) $$A_{\mathrm{ij}}=\frac{X_{\mathrm{ij}}-X_{\min}}{X_{\max}-X_{\min}}$$

For negative indicators which means that the smaller the value of the indicator the better, the formula for normalising negative indicators is as follows:

(2) $$A_{\mathrm{ij}}=\frac{X_{\max}-X_{\mathrm{ij}}}{X_{\max}-X_{\min}}$$

Where *A*_*ij*_ denotes the standard value of indicator *j* for sample *i*, *X*_*ij*_ is the actual value of indicator *j* for sample *i*, *X*_min_ denotes the minimum value of indicator *j* and *X*_max_ denotes the maximum value of indicator *j*.

Indicators are standardised to have a value domain interval of [0, 1] and are all positive indicator values, that is, the larger the value, the better it is for regional ecological security.

The key issue of ecological security evaluation is the weight calculation of indicators, and the reasonable weight has a great influence on the results of ecological security evaluation. At present, the commonly used weight calculation methods include the hierarchical analysis method, entropy value method and so on. The hierarchical analysis method is used to determine the advantages and disadvantages of the program by comparing the relevant factors at the same level horizontally and then vertically between different levels (Seyedmohammadi et al., 2019), which has been widely used in ecological security evaluation research as a commonly used subjective assignment method (He et al., 2021). The entropy weight method is an objective empowerment method, that judges the degree of orderliness of a system by measuring the information entropy of the system, and the higher the information entropy of an evaluation index in ecological security evaluation, the higher the status of the index in the evaluation system and the higher the weight value (Gao et al., 2006). This study adopts the combination of hierarchical analysis method and entropy weighting method to assign weights, and establishes an optimisation decision model by introducing the Lagrangian function to ensure the consistency between the subjective and objective weights and preference coefficients, and then obtains the scientific combined weights of the indicators (Zhu & Zhang, 2017), and the formula of the combined weights is as follows, and the weights of the indicators of PLES in Yunnan Province are shown in Table 4.

(3) $$D\left(W_{\mathrm{aj}}-W_{\mathrm{bj}}\right)=\sqrt{\sum_{j=1}^{n}{\left(W_{\mathrm{aj}}-W_{\mathrm{bj}}\right)}^{2}}$$

(4) $$D{\left(W_{\mathrm{aj}}-W_{\mathrm{bj}}\right)}^{2}={\left(\alpha -\beta \right)}^{2}$$

(5) α+β=1

(6) $$W_{j}=\alpha W_{\mathrm{aj}}+\beta W_{\mathrm{bj}}$$

**TABLE 4:** Combined weights of indicators for evaluating ecological security of PLES in Yunnan province.

Production space				Living space				Ecological space			
Indicator	AHP	Entropy weight	Combined weight	Indicator	AHP	Entropy weight	Combined weight	Indicator	AHP	Entropy weight	Combined weight
PSP1	0.0665	0.2013	0.1142	LSP1	0.0674	0.0102	0.0472	ESP1	0.0464	0.0189	0.0367
PSP2	0.0719	0.0348	0.0588	LSP2	0.0603	0.0217	0.0466	ESP2	0.2040	0.0531	0.1506
PSP3	0.0923	0.0026	0.0605	LSP3	0.1912	0.0091	0.1267	ESP3	0.1273	0.0799	0.1105
PSP4	0.0914	0.0082	0.0619	LSS1	0.1125	0.0174	0.0788	ESS1	0.0594	0.0900	0.0702
PSP5	0.0635	0.0375	0.0543	LSS2	0.1353	0.0364	0.1003	ESS2	0.0607	0.0388	0.0529
PSS1	0.0853	0.0251	0.0640	LSS3	0.0500	0.1104	0.0714	ESS3	0.0521	0.0291	0.0440
PSS2	0.1093	0.0635	0.0931	LSS4	0.0942	0.1102	0.0999	ESS4	0.0556	0.0815	0.0648
PSS3	0.1317	0.3230	0.1994	LSS5	0.0461	0.1143	0.0702	ESS5	0.0562	0.0311	0.0473
PSR1	0.1475	0.1069	0.1331	LSR1	0.0720	0.0757	0.0733	ESS6	0.0937	0.0511	0.0786
PSR2	0.0845	0.0888	0.0860	LSR2	0.0445	0.1304	0.0749	ESR1	0.1666	0.3732	0.2397
				LSR3	0.0304	0.0707	0.0447	ESR2	0.0778	0.1533	0.1045
				LSR4	0.0375	0.0945	0.0577				
				LSR5	0.0585	0.1992	0.1083				

*W*_*aj*_ is the average weight determined by hierarchical analysis, *W*_*bj*_ is the average weight determined by the entropy weighting method, *α* is the coefficient of the degree of subjective preference, *β* is the coefficient of the degree of objective preference, and *W*_*j*_ is the combined weight.

#### 3.3.3 Comprehensive evaluation and classification

The ecological security status is measured by weighted summation after standardisation of each indicator, based on the comprehensive ecological security value obtained, and the calculation formula is shown in formula (7). The ecological security assessment value is not conducive to the visual expression of the ecological security status, and it is usually graded based on the ecological security evaluation value, which is a necessary and important step in the ecological security evaluation. Ecological security is affected by natural, social, economic and other factors, and has complexity and relativity. Currently, relevant studies have not formed a uniform ecological security grading standard (Xie & Li, 2004). In our study, based on the actual situation of Yunnan Province, taking into account domestic and international studies (Chen & Wang, 2020), and comprehensively analysing the values of PLES ecological security evaluation results in Yunnan Province, we determined the grading standard of PLES ecological security index in Yunnan Province by using the method of equal intervals combined with the natural breaking point method. The grades were determined as five levels, that is, Unsafe, Relatively unsafe, Critical safe, Relatively safe and Safe, and the grading standards and characteristics of ecological security at all levels are shown in Table 5.

(7) $$\mathrm{ESI}=\sum_{j=1}^{n}W_{j}\times X_{j}$$

**TABLE 5:** Criteria and characteristics of ecological security grade.

Class	Ecological security characteristics of production space	Ecological security characteristics of living space	Ecological security characteristics of ecological space
Unsafe	[0–0.28] Ecosystems are fragile, severely damaged and highly vulnerable to ecological disasters, and there is little human response to the problems	[0–0.35] Agricultural production is seriously threatened, soil quality is degraded, crop damage is severe, and human response is minimal	[0–0.38] There are serious problems of pollution and damage to water, air and noise, and the quality of residents' livelihoods has deteriorated
Relatively unsafe	[0.28–0.33] Ecosystems are relatively fragmented and damaged to a relatively high degree, with a higher potential for disasters and less human response	[0.35–0.41] Agricultural production has relatively serious problems with pesticide residues and water stress	[0.38–0.48] There are relatively problems with water, air and noise which may have an impact on the health and quality of residents' livelihoods
Critical safe	[0.33–0.38] The ecological system is structurally unstable and may experience ecological problems when disturbed, and is in a critical state between security and insecurity	[0.41–0.47] It is in a critical state of safety and insecurity, with problems of dependence on chemical fertilisers and pesticides and limited crop yields	[0.48–0.58] A critical state of security and insecurity, with problems of land, water and energy development and utilisation
Relatively safe	[0.38–0.43] Ecosystems are more stable in terms of broken structure and functioning, with few ecological problems and more protective measures in place	[0.47–0.53] Production ecological security is better protected and repaired, crop quality and yield are improved, and humans are aware of and take measures to protect it	[0.58–0.68] Living space ecosystems are better protected and restored, and environmental quality has improved
Safe	[0.43–1] The ecosystem is stable and it is highly resistant and resilient	[0.53–1] Agricultural production is not only economically efficient but also ecologically efficient, with a high degree of human protection	[0.68–0.1] Protection and development are optimally balanced, and the living environment and health of the population are adequately safeguarded

ESI regional comprehensive ecological security value, *W*_*j*_ is the combined weight of the *j*th indicator and *X*_*j*_ is the value of the *j*th indicator. The larger the ESI value, the better the ecological security status, on the contrary, the worse the status.

## 4 RESULTS

### 4.1 Spatial patterns of PLES

**FIGURE 3:**
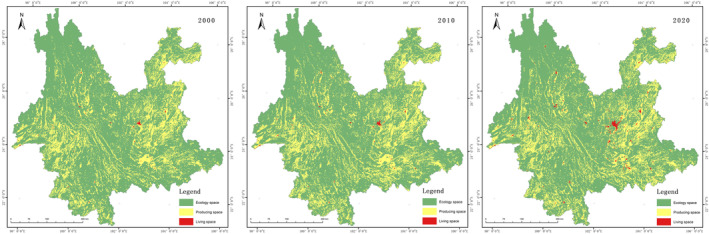
Results of PLES in Yunnan Province.

The spatial structure of PLES in Yunnan Province is dominated by ecological space, accounting for more than 75% of the total, with production space accounting for 23% and living space accounting for <1%, as shown in Figure 3, and the statistics of the area and the percentage of the area are shown in Table 6. Production space is concentrated and continuous in the Hengduan Mountains in western Yunnan, Pu'er and Xishuangbanna in southern Yunnan, and the Wuliang Mountains in central Yunnan, and more fragmented in eastern and north‐eastern Yunnan. Production space is mainly distributed in the dam areas of central, southern and western Yunnan, as well as in other areas with relatively low topographical undulations. The living space is mainly distributed in the dams of the urban agglomeration in central Yunnan and the town locations of Dali and Baoshan in western Yunnan. The area of ecological space showed a decreasing trend from 2000 to 2020, while both productive and living space increased.

**TABLE 6:** PLES area statistics in Yunnan Province.

Year	Production space		Living space		Ecological space	
	Area (km^2^)	Proportion (%)	Area (km^2^)	Proportion (%)	Area (km^2^)	Proportion (%)
2000	88946.95	22.57	778.72	0.20	304374.33	77.23
2010	94067.41	23.87	1063.64	0.27	298968.95	75.86
2020	94235.71	23.91	2683.09	0.68	297181.20	75.41

The spatial distribution of PLES in Yunnan Province in different periods was analysed by geographical information software. From 2000 to 2020 it is mainly ecological space that converted into production space, mainly in Xishuangbanna and southern Honghe Prefecture in southern Yunnan. The spatial distribution from 2010 to 2020 is still more from ecological space to the production space, with an increase in western Yunnan in addition to southern Yunnan. The areas where ecological space is transformed into living space are mainly located around urban areas such as Kunming and Qujing.

### 4.2 Ecological security based on county scale

#### 4.2.1 County‐scale ecological security of production spaces

**FIGURE 4:**
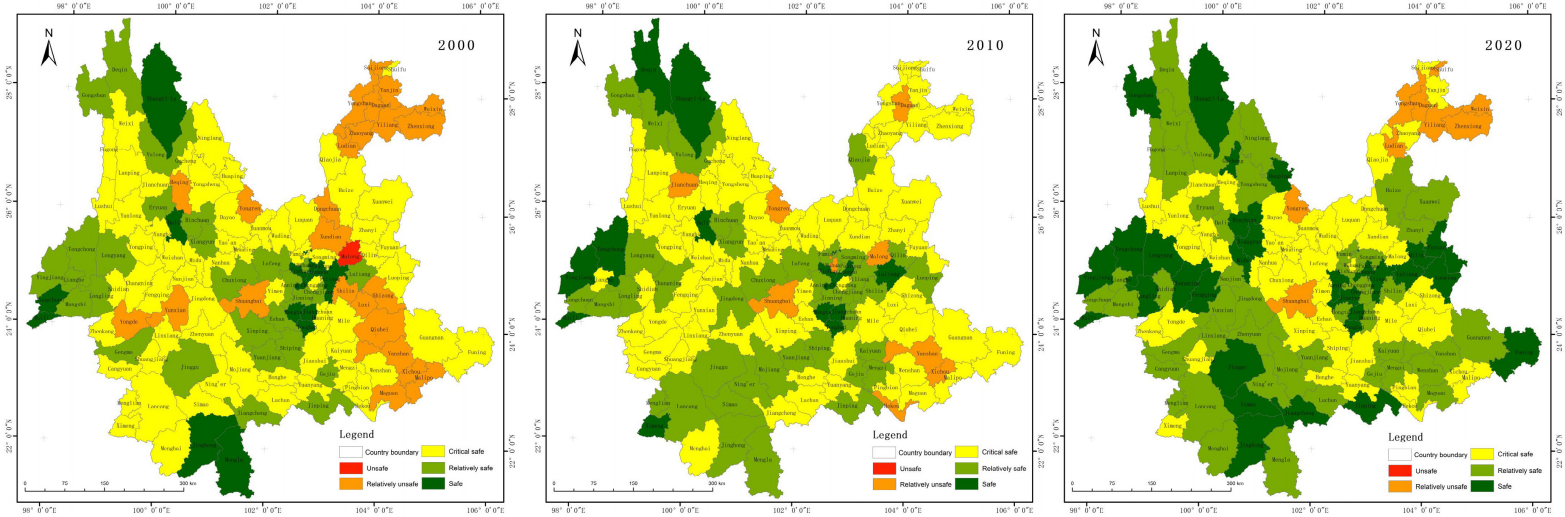
Maps of production space ecological security on County‐scale.

The results of the ecological security evaluation of the production space shown in Figure 4. The pattern of ecological security in 2000 was better in the west than in the east, with most counties in the critical safe. 2010 saw an improvement compared to 2000, and the pattern of distribution was generally consistent, with the unsafe areas decreasing in central and north‐eastern Yunnan. 2020 saw the distribution of safe class stabilise and increase significantly in eastern Yunnan and western Yunnan, but decrease in Zhaotong in north‐eastern Yunnan.

#### 4.2.2 County‐scale ecological security of living spaces

**FIGURE 5:**
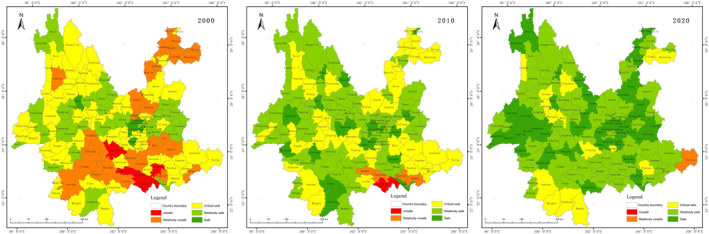
Maps of living space ecological security on County‐scale.

The results of the ecological security evaluation of living space are shown in Figure 5. The pattern of ecological security in 2000 was better in the centre than in the surrounding area. The safe class mainly located in the main urban area of Kunming City, and unsafe class roughly divided into two areas located in Zhaotong, northeastern Yunnan Province, and southern Yunnan Province. 2010 saw an increase in the sense of overall security compared to 2000, with the unsafe class drastically reduced to only the border of southern Yunnan Province, and an increase in safe class in the state and municipal government areas. 2020 will see a shift to a better overall situation in terms of distribution. The pattern remains stable.

#### 4.2.3 County‐scale ecological security of ecological spaces

**FIGURE 6:**
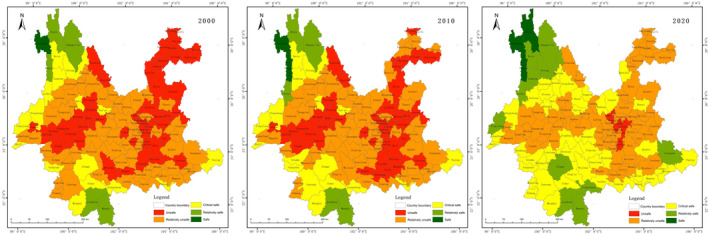
Maps of ecological space ecological security on County‐scale.

The results of the ecological security evaluation of the ecological space are shown in Figure 6. In 2000, ecological security was dominated by unsafe class and relatively unsafe class, and safe class was distributed only in north‐western Yunnan and southern Yunnan. In 2010, the pattern was consistent with that of 2000, and the distribution of unsafe class was reduced. In 2020, the ecological security was greatly improved, dominated by critical safe and relatively unsafe, and the unsafe class was drastically reduced to be distributed only in Kunming.

### 4.3 PLES ecological security based on grid‐scale

#### 4.3.1 1 km × 1 km grid scale ecological security of production space

**FIGURE 7:**
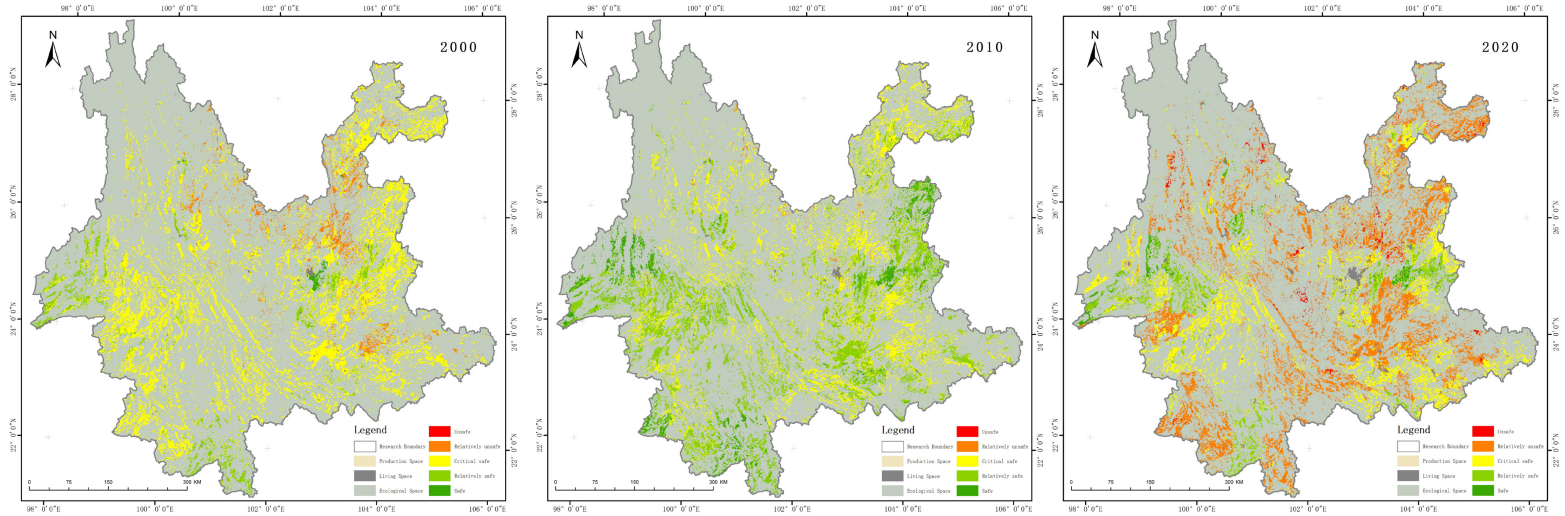
Maps of production space ecological security on Grid‐scale.

The results of the ecological security evaluation of the production space at the grid scale are shown in Figure 7. The ecological security of productive space in 2000–2020 shifted from critical safe to relatively safe and then to unsafe. The ecological security rating is dominated by critical safe and relatively safe in 2000 and 2010, and critical safe and relatively unsafe in 2020.

In 2000, ecological security was dominated by critical safe, accounting for 78.97%, which was widely distributed throughout the province. The relatively unsafe accounted for 10%, mainly in Dehong Prefecture in north‐western Yunnan and Xishuangbanna Prefecture in southern Yunnan. The relatively safe accounted for 10%, mainly distributed in Kunming in central Yunnan and Wenshan in western Yunnan and eastern Honghe Prefecture in southern Yunnan. Ecological security improved significantly in 2010. The safe class increased from 0.5% to 7.41%, mainly in Qujing in northeastern Yunnan, Dehong in western Yunnan and Pu'er in southern Yunnan. The relatively safe class rose from 11.42% to 48.11%, mainly in Baoshan and Lincang in western Yunnan and Qujing in eastern Yunnan. Ecological security declines significantly in 2020. Relatively unsafe jumped significantly to 49.14%, mainly in Zhaotong and Qujing in northeast Yunnan, central Yunnan and Pu'er and Xishuangbanna in south Yunnan. Critical safe accounts for 34.37%, mainly in northern Yunnan Pu'er, eastern Lincang and southern Honghe Prefecture. Relative safe accounted for 12.58%, mainly in Dehong Prefecture and western Baoshan City in western Yunnan and southern Qujing in north‐eastern Yunnan.

#### 4.3.2 1 km × 1 km grid scale ecological security of living space

**FIGURE 8:**
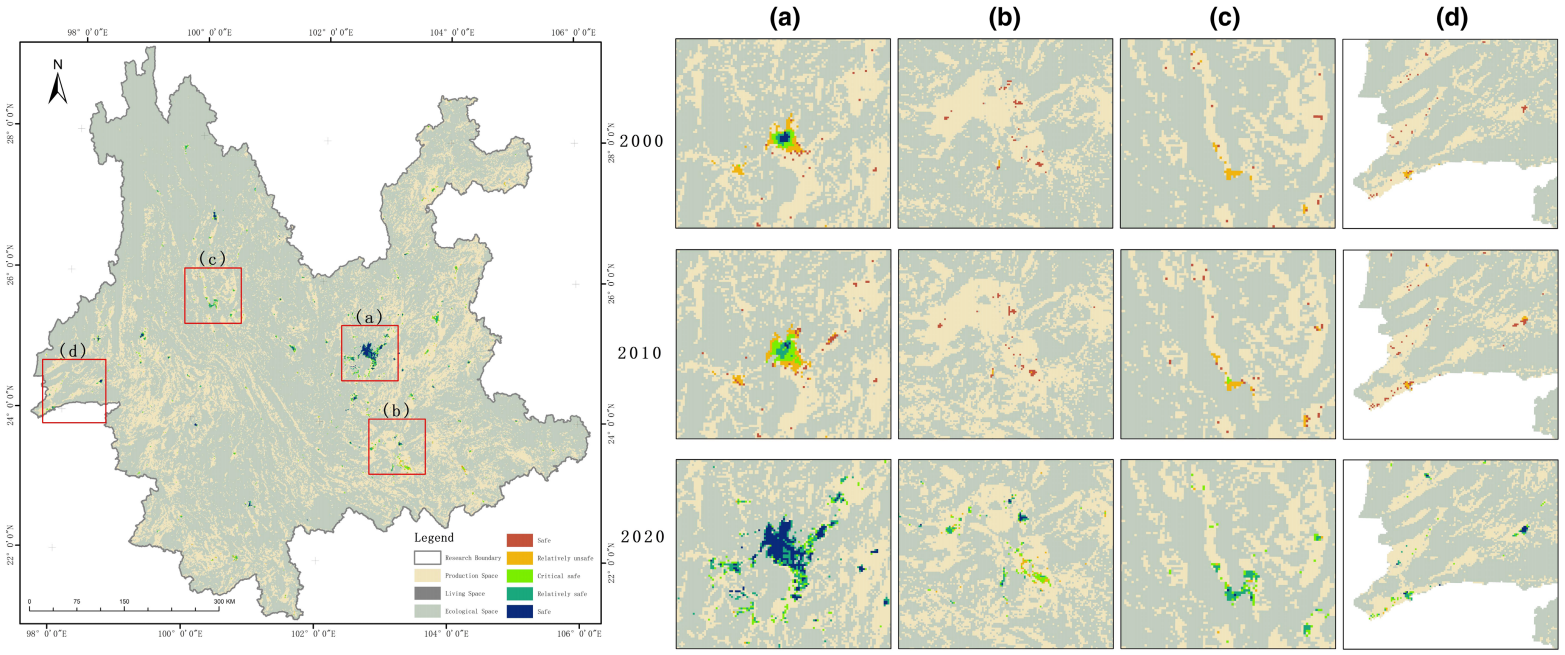
Maps of living space ecological security on a Grid‐scale.

The results of the ecological security evaluation of the Living space at the grid scale are shown in Figure 8. Unsafe and relatively unsafe predominated in 2000. The safe class was only 10%, distributed in Kunming. The unsafe class was distributed throughout the province, accounting for 58.33%. The relatively unsafe class were mainly in the centre. Overall in 2010, there was a slight improvement from 2000. The distribution pattern is basically the same as in 2000. The ecological security situation will be significantly improved in 2020. It is dominated by critical safe and relatively safe, mainly concentrated in Kunming, Yuxi, Baoshan and Dehong.

#### 4.3.3 1 km × 1 km grid scale ecological security of ecological space

**FIGURE 9:**
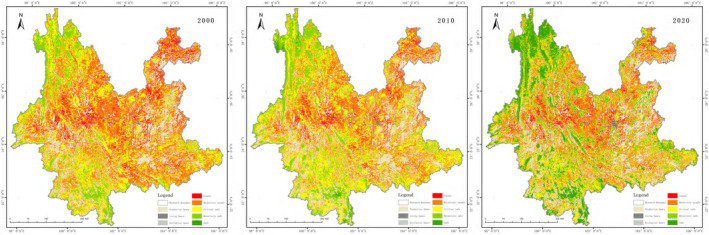
Maps of ecological space ecological security on Grid‐scale.

The results of the ecological security evaluation of the ecological space at the grid scale are shown in Figure 9. Overall, relatively unsafe and relatively safe predominate between 2000 and 2020, the proportion of relatively unsafe declines, and the proportions of critical safe, relatively safe, and safe increase.

**FIGURE 10:**
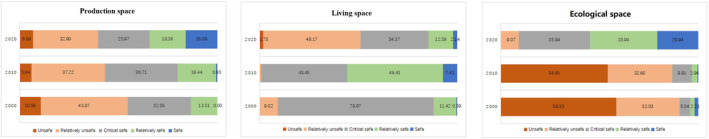
Map of the percentage of ecological security levels in the PLES.

In 2000, relatively unsafe accounted for 43.87%, mainly in central Yunnan, northeastern Yunnan and south‐eastern Yunnan. Critical safe accounted for 32.05%, mainly in Pu'er, Banna and Lincang in southern Yunnan. Relatively safe accounted for 13.51%, mainly distributed in Diqing and Nujiang in north‐western Yunnan and Xishuangbanna in southern Yunnan. Unsafe accounts for 10.56%, mainly in Kunming in central Yunnan and Dali in western Yunnan. In 2010, there was an overall improvement over 2000, with the level of unsafe improving in northeastern Yunnan. Relatively unsafe improved in central Yunnan. Critical safe improved in southeast Yunnan. The distribution of the relatively safe is basically the same as that of 2000. In 2020, the distribution of the overall pattern remains essentially the same. Unsafe and critical safe are reduced from 2010. The relatively safe distribution changes from contiguous to scattered. The increase in the safe class is mainly shifted by the safer areas in north‐western Yunnan and Xishuangbanna in southern Yunnan. The area and percentage of each class of ecological security of PLES 2000, 2010 and 2020 in Yunnan Province are shown in Table 7 and Figure 10.

**TABLE 7:** Area and percentage of graded results of the evaluation of ecological security of PLES in Yunnan province.

Class		Production space			Living space			Ecological space		
		2000	2010	2020	2000	2010	2020	2000	2010	2020
Unsafe	Area (km^2^)	17,206	19,576	19,576	0	0	1622	448	567	3
	Per cent (%)	5.84	6.68	6.68	0.00	0.00	1.75	58.33	54.05	0.11
Relatively unsafe	Area (km^2^)	109,733	96,429	96,429	7912	676	45,695	246	342	240
	Per cent (%)	37.22	32.90	32.90	9.02	0.73	49.17	32.03	32.60	9.07
Critical safe	Area (km^2^)	108,246	76,104	76,104	69,272	40,309	31,942	41	104	951
	Per cent (%)	36.71	25.97	25.97	78.97	43.45	34.37	5.34	9.91	35.94
Relatively safe	Area (km^2^)	57,322	53,814	53,814	10,021	44,916	11,689	17	31	898
	Per cent (%)	19.44	18.36	18.36	11.42	48.41	12.58	2.21	2.96	33.94
Safe	Area (km^2^)	2348	47,150	47,150	517	6872	1985	16	5	554
	Per cent (%)	0.80	16.09	16.09	0.59	7.41	2.14	2.08	0.48	20.94

## 5 DISCUSSION

Based on the results of the division of PLES, this study innovatively constructed a sub‐district ecological security evaluation model for Yunnan Province given the functional characteristics of different national land spaces, and evaluated the ecological security of PLES in Yunnan Province at both county and grid scales for the periods of 2000, 2010 and 2020. Through this study, we can find the spatial distribution characteristics and temporal evolution pattern of ecological security in PLES in Yunnan Province, and make new explorations for the further exploration of ecological security characteristics and ecological security maintenance measures under different territorial functional zones, which will play an important role in the formulation of regional ecological environmental protection strategies, guidance of territorial spatial development and orientation of sustainable development.

### 5.1 Evaluation model and indicator system for ecological security of PLES

The ecological security evaluation used in this study uses the PSR framework, which is one of the more mature and widely used models in ecological security evaluation. This study focuses on the characteristics and attributes of productive, ecological, and living spaces under the PSR framework, and analyses the state of pressure and response faced by regional ecological security from the perspective of sustainable development.

Based on the analysis of the dominant function of living space, the main pressure comes from rapid population growth, land resources shortage, environmental degradation, etc., characterising the state of living space as the state of socio‐economic development, the area of construction land, educational resources, medical resources, transportation resources, etc., and the response and regulation can be carried out in terms of urban construction, improvement of public services, pollution control, etc. (Chen et al., 2016; Guo et al., 2020; Huang et al., 2016; Xie & Li, 2004).

Based on the analysis of the dominant function of the production space, the main pressures facing the production space come from rapid population growth, land resource shortage, frequent natural disasters, water resource shortage, soil pollution, etc.; the state of the production space is characterised by the arable land area, the quality of arable land, the regional cultivation water and heat conditions, the quality and safety of agricultural products, the pesticide and chemical fertiliser residuals, and the production value of major crops; the response and regulation can be achieved through land remediation, land rotation, agricultural planting structure adjustment, water‐saving irrigation, soil pollution control, and pesticide and fertiliser reduction (Li, 2011; Liu et al., 2021; Tao & Luo, 2004; Wang et al., 2014; Wu, 2001; Xu et al., 2021).

Based on the analysis of the dominant function of ecological space, the main pressure faced by ecological space comes from human activity interference, climate change, environmental degradation, natural disasters, etc., and the state of the ecological space is characterised by the state of the vegetation cover, landscape pattern and carbon storage, and the response and regulation can be carried out from the aspects of planting forests, soil and water erosion control, rocky desertification control, and the establishment of protected areas, etc. (Fan & Fang, 2020; Li et al., 2020; Tang et al., 2020; Wu et al., 2019).

The ecological security evaluation model of PLES spatial zoning constructed in this study is a new exploration for ecological security evaluation, and the model is also more capable of reflecting the characteristics of regional ecological security and the problems that need to be considered. It is also an innovative combination of ecological security evaluation and functional zoning of national land space, which is of great significance to the identification of problems, coordination and optimisation of the pattern of national land space, and provides scientific solution ideas for achieving regional sustainable development.

### 5.2 Problems of evaluating the scale of ecological security in PLES

In our study, we did two scales of ecological security evaluation studies in the county and 1 km grid, for the exploration of ecological security evaluation changes in different scales, which can better reveal and understand the spatial heterogeneity, and adaptive analysis for specific areas or specific problems. Research at the county scale helps government departments and related organisations to fine‐tune their management and decision‐making on ecological environmental protection, and to provide more specific suggestions and strategies for local planning, construction and development.

The grid‐scale study can analyse the social, economic and environmental influences on ecological security in the region in more detail, and can better reflect the details of the regional spatial pattern. The results of this study show that changes in ecological security at the county scale are strongly influenced by socioeconomic development, while changes in ecological security at the grid scale are mainly influenced by natural conditions. The main influences on the differences in the distribution of ecological security of the production space at the grid scale are topography and precipitation, while at the county scale, they are influenced by factors such as the value of production and irrigation conditions. Living space ecological security at the county scale mainly reflects macro‐regional development differences, while at the grid scale one can see ecological security differences between large and small cities as well as regional differences within cities. Ecological security in ecological space is better reflected at the county scale in terms of differences in the situation of human inputs to ecological protection and at the grid scale in terms of the details of the distribution of patterns. At present, scholars of ecological security studies determine the scale of study based on the scope of the study area, and most of them are based on administrative areas, grids and watersheds, for example, Chen et al. (2022) conducted an ecological security evaluation study of the county and grid in the central Yunnan area, and Tang et al. (2020) carried out an ecological security study in the Chaohu Basin, and there are also studies on ecological security studies based on the landscape and ecosystem scales (Shi et al., 2021); however, there is still a relative lack of discussion on ecological security studies at different scales of study.

Zuo et al. (2003) investigated the discriminatory relationship between region, mapping, visualisation of mapping, data accuracy and data load in determining the size of the evaluation unit of the regional grid (Zuo et al., 2003). Ju  et al. (2020) argued that ecological security issues at different scales have different emphases, and at the same time, there are mutual containment relationships and material, energy and information connections. In the future, we can deepen the research on multi‐scale ecological processes by strengthening the research on the correlation mechanism of ecological risks among scales, so that the scale relationship can become a more reliable theoretical basis for ecological security (Ju et al., 2020).

### 5.3 Spatial and temporal consistency of ecological security in PLES

In the study on the functional characteristics and zoning optimisation of the PLES in Yunnan Province by Li et al. (2021), the ecological function advantageous area accounted for the largest proportion and was mainly concentrated in the western part of Yunnan Province, while the production‐living function area accounted for the second largest proportion and was mainly expanded outward with central Yunnan as the core, and was concentrated in the urban agglomeration within the scope of central Yunnan, which is consistent with the conclusions of the PLES spatial distribution and ratio in our study (Li et al., 2021).

Chen et al. (2022) on the evaluation of ecological security in the central Yunnan region showed that the ecological security in the central Yunnan region from 2005 to 2015 showed an upward trend, which is consistent with the conclusions of this study that the ecological security of the ecological space from 2010 to 2020 has improved, followed by the results of Chen's study that the ecological security of the central, southern, and eastern parts of the central Yunnan region has improved more significantly which is also consistent with the conclusions of this study (Chen et al., 2022). Xie et al. (2023) compared the results of Yunnan Province's ecological security evaluation study, and the province's ecological security showed an improving trend is also consistent with the findings of this study (Xie et al., 2023). The results of this study were compared with the existing related research results and came to a consistent conclusion, which further verified the scientificity and validity of this study. This means that our research method can be accepted and applied, which is of great significance for further research and practice in related fields such as PLES and ecological security.

### 5.4 Limitation and prospect

In the study, the classification method used for the division of PLES is the combination of land classes, which cannot better reflect the complex multifunctionality of land, the complexity of spatial functions, and the heterogeneity of the substrate, and it is difficult to characterise the qualitative differences in the national land space, and the results are subject to the influence of the experts' a priori knowledge and the classification system. The results are affected by experts' a priori knowledge and the classification system, with certain subjectivity and relatively low accuracy, etc. In the future, we can consider improving the comprehensiveness of the division of PLES through methods such as the quantitative measurement method of national land space.

Although the ecological security of different regions has been investigated in this study in terms of production, ecological and living space, this part of the study has not been explored deeply enough due to the complex topography and geomorphology of Yunnan Province, as well as the special climate that leads to different factors influencing the ecological security of different regions.

The ecological security factors of PLES in Yunnan Province can be analysed in the future through geographical probes, geographical weighted regression and other methods. The ultimate goal of ecological security evaluation is to realise the sustainable development of the region, so that regional ecological security can be maintained at a better level, and the maintenance of regional ecological security can be further explored in the future through the construction of ecological security pattern and the calculation of ecological obstacle degree factors.

## 6 CONCLUSION

In this study, the PLES ecological security diagnostic index system was established from the perspective of the function of the national land space, which solves the problem that it is difficult to comprehensively and accurately reflect the state of ecological security at the regional scale, and realises the quantitative evaluation of regional ecological security in the functional dimension. The conclusions are as follows:

1. The spatial pattern of PLES is dominated by ecological space, and the ratio of ecological production and living is 7.5:2.5:1. In the past 20 years, the ecological space has shrunk, and the production space and living space have shown a trend of expansion, reflecting the characteristic of Yunnan Province that is dominated by ecological functions. The scarcity of land resources has led to the persistent contradiction between ecological protection and development.
2. The ecological security of the production space showed an improvement in 2010 compared with 2000, but the ecological security of the production space in 2020 will decline again sharply. The areas with a safe level of production space are mainly located in Dehong Prefecture in western Yunnan and Xishuangbanna in southern Yunnan, where water and heat conditions are favourable for production. Areas rated as unsafe are mainly located in Kunming and northern Honghe Prefecture in central Yunnan. The ecological security of the production space is characterised by a clear administrative district. Yunnan Province has relatively favourable background conditions for agricultural production, but there are large regional differences, and there is still a need to improve management and investments.
3. The ecological security of living space has shown an improving trend in the past 20 years. The areas rated as safe are mainly located in Kunming City in central Yunnan Province, which, as the capital city of Yunnan Province, has better infrastructures and governmental inputs, so the ecological security of living space is relatively more comprehensive. The spatial distribution of ecological security in the living space is characterised by the superiority of the east over the west and the south over the north. County‐scale studies reveal imbalances in regional development, but regional disparities in the province have been gradually narrowing over the past 20 years. The grid‐scale results reflect a better level of ecological security at the location of political sites.
4. The ecological security of Yunnan Province's ecological space showed an improving trend from 2000 to 2020, and the change between 2000 and 2010 is smaller than the difference between 2010 and 2020. The areas with ecological security are mainly located in north‐western Yunnan and southern Yunnan, which are ecologically sensitive and fragile areas in Yunnan Province, and also areas where ecological protection measures are more comprehensively implemented and better realised. Ecological security is mainly distributed in central Yunnan, north‐eastern Yunnan, and south‐eastern Yunnan. Central Yunnan is an area where the contradiction between socio‐economic development and ecological protection is more prominent, while the ecological security of north‐eastern Yunnan and south‐eastern Yunnan is limited by natural conditions such as topography and climate, and it is still necessary to strengthen the ecological protection measures and inputs according to the local conditions in these areas.

## AUTHOR CONTRIBUTIONS

**Fang Liu:** Conceptualization (equal); data curation (equal); funding acquisition (equal); writing – original draft (equal); writing – review and editing (equal). **Qian Zhang:** Formal analysis (equal); methodology (equal); resources (equal); writing – original draft (supporting). **Jinliang Wang:** Conceptualization (equal); formal analysis (equal); funding acquisition (equal); supervision (equal). **Yuexiong Liu:** Resources (equal); software (equal); visualization (equal). **Wanbin Wang:** Investigation (equal); methodology (equal); software (equal); validation (equal). **Sen Li:** Investigation (equal); project administration (equal); supervision (equal).

## CONFLICT OF INTEREST STATEMENT

The authors declare that they have no known competing financial interests or personal relationships that could have appeared to influence the work reported in this paper.

## Data Availability

The data that supports the findings of this study are available in the Supporting Information of this article.
